# Benchmark suites instead of leaderboards for evaluating AI fairness

**DOI:** 10.1016/j.patter.2024.101080

**Published:** 2024-11-08

**Authors:** Angelina Wang, Aaron Hertzmann, Olga Russakovsky

**Affiliations:** 1 Princeton University, Princeton, NJ, USA; 2 Stanford University, Stanford, CA, USA; 3 Adobe Research, San Francisco, CA, USA

## Abstract

Benchmarks and leaderboards are commonly used to track the fairness impacts of artificial intelligence (AI) models. Many critics argue against this practice, since it incentivizes optimizing for metrics in an attempt to build the “most fair” AI model. However, this is an inherently impossible task since different applications have different considerations. While we agree with the critiques against leaderboards, we believe that the use of benchmarks can be reformed. Thus far, the critiques of leaderboards and benchmarks have become unhelpfully entangled. However, benchmarks, when not used for leaderboards, offer important tools for understanding a model. We advocate for collecting benchmarks into carefully curated “benchmark suites,” which can provide researchers and practitioners with tools for understanding the wide range of potential harms and trade-offs among different aspects of fairness. We describe the research needed to build these benchmark suites so that they can better assess different usage modalities, cover potential harms, and reflect diverse perspectives. By moving away from leaderboards and instead thoughtfully designing and compiling benchmark suites, we can better monitor and improve the fairness impacts of AI technology.

## The bigger picture

As artificial intelligence (AI) systems become increasingly complex and ubiquitous, developers and users need to understand these systems’ social fairness impacts: their potential for harm to different communities. Fairness is a debated concept, and constructing measurements of social fairness is challenging. Doing so requires considering different applications, capturing different types of harms, and reflecting different communities’ perspectives. Hence, the common approach of scoring models by AI fairness leaderboards, which attempt to reduce fairness to a single number, is inappropriate. Instead of measuring single scores, we argue for comprehensive benchmark suites, which expose multiple measurements, allowing researchers and practitioners to better understand the different trade-offs between AI models, without hiding potential harms within a single score. We describe principles for the construction and use of such suites.

## Teaser

The authors argue for the elimination of leaderboards in artificial intelligence (AI) fairness, because “fairness” cannot be measured as a single number. Instead, they propose the development and curation of benchmark suites, which allow separate monitoring of distinct fairness considerations. Benchmark suites enable researchers and practitioners to better understand the various fairness impacts of AI technology that will be relevant in different ways in different applications.

## Introduction

How do we understand the potential social harms and inequities of artificial intelligence (AI) models?^1^^,^^2^^,^^3^ One way that AI fairness researchers have responded to this question is by creating benchmarks. Each benchmark includes a dataset and associated metric,^4^ intending to measure a particular dimension of AI fairness. As an example, the Bias Benchmark for QA (BBQ) uses multiple-choice questions to measure language models’ bias toward different demographic groups by querying the model with questions such as about whether a boy or a girl more likely struggles with math.^5^

A common practice when developing new algorithms is to report scores on leaderboards. A leaderboard ranks algorithms based on a single score that is either computed over one benchmark or aggregated over multiple benchmarks. These rankings exist on dynamic websites determining the state-of-the-art,^6^^,^^7^^,^^8^^,^^9^ as well as in the tables of academic papers to establish superiority over prior methods. Technical research agendas sometimes focus on building AI models that top a leaderboard.

Many recent authors criticize the use of benchmarks and leaderboards within fairness research.^10^^,^^11^^,^^12^ They rightly point out that different harms cannot be meaningfully averaged into a single score, and moreover, benchmark optimization encourages “gaming” the metrics. These points echo concerns across AI in general regarding the use of benchmarks and leaderboards.^4^^,^^13^^,^^14^^,^^15^^,^^16^^,^^17^^,^^18^

Nonetheless, benchmarks can be quite useful for identifying potential harms and operationalizing ethical principles.^19^ Indeed, some of the most impactful fairness changes in AI models have come from quantitative measurements (e.g., Gender Shades,^20^ COMPAS^21^). Arguably, many of the criticisms of benchmarks are really criticisms of leaderboards. Can we use benchmarks productively without leaderboards?

We argue that fairness benchmarks should be used not for leaderboards, but rather collected into “benchmark suites” that provide insights into a model’s trade-offs around a set of potential harms. Benchmark suites, as we call them, are collections of individual benchmarks, consisting of multiple datasets and metrics. While these collections are sometimes referred to as benchmarks as well, we reserve the term “benchmark” for individual measurements. Recent examples for large language models (LLMs) that fall under our definition of a benchmark suite include DecodingTrust,^22^ TrustLLM,^23^ and HELM,^24^ as well as the set of evaluations used in model cards like Claude 3’s^25^ and Llama’s.^26^ However, despite being collections of benchmarks, these collections do not meet what we will present as best practices for benchmark suites.

We propose that a suite should be curated with the goal of completeness in capturing the construct of interest. Suites can then serve as a guiding tool for selecting among all of the proliferating benchmarks. The results of applying these suites can provide researchers and practitioners ways to analyze the behavior of their models, anticipate potential problem areas, and understand the corresponding trade-offs. For example, consider the task of understanding the ability of an LLM to select merit-based scholarship recipients. One LLM might make a race-blind decision and another may take a race-aware approach. Separate benchmark metrics can expose this trade-off, while other metrics might reveal effects relating to gender and intersectional dynamics. The preferred model, if any at all, will depend on the deployment scenario, for example, whether the scholarship is diversity based or whether race-blind legislation applies to the scholarship. A benchmark suite here would help to proactively identify potential discrimination harms and trade-offs between models, without determining one model to be categorically better than another.

We need to understand, however, how this idea fits within the standard paradigm of technical AI research, which focuses on optimizing for and comparing models against leaderboards. Here, we present constructive ways that benchmark suites can be used in lieu of leaderboards toward the goals of making research progress and selecting models for deployment.

Making benchmark suites effective for these uses requires further research to improve them. We suggest three necessary improvements toward this goal: creating benchmarks specific to individual modalities, systematically analyzing harm coverage and limitations, and representing diverse perspectives. In doing so, we put forth benchmark suite curation as a critical research direction.

Overall in this work, we focus on examples and recommendations specifically attuned for the fairness setting, but many of these arguments generalize to measurement in AI at large.

## The problem with fairness leaderboards

Many have criticized the use of leaderboards to rank fairness algorithms.^13^^,^^14^^,^^27^ Leaderboards for a single benchmark misleadingly use one measurement to capture the entire notion of fairness. Even leaderboards for a collection of benchmarks report rankings based on a single score aggregated over the whole collection, so that an algorithm may give an improved “fairness score” by increasing performance on one benchmark while making it worse on another. However, one benchmark might be assessing a very different potential harm from another; for some applications, this apparent improvement could actually be much worse.

For example, an image captioning algorithm can be used for different applications, such as indexing image search results, or assisting blind and low-vision users. Some blind and low-vision users have expressed a desire for detailed image descriptions that may include inferred gender because that provides useful social context for understanding an image.^28^ However, transgender and non-binary individuals may object to automated gender classification because of the dignitary harms of being misgendered or the reification of rigid gender categories.^29^^,^^30^ A leaderboard averaging two different measurements, one around accurate gender inference and one around the lack of any gender inference, would obscure these tensions and be counterproductive.

This points to a fundamental issue: whether one model is preferred over another depends on the context in which it is used and the ethical values that are prioritized in that context.^31^ Hence, during research, different fairness considerations cannot be meaningfully summarized with a single score, making leaderboards misleading. Pareto improvements (i.e., the notion that one model can, in all respects, match or outperform another model) will often be impossible under well-constructed suites of fairness benchmarks because some measurements will directly conflict. A related scenario can be seen in the famous fairness impossibility theorems, which demonstrate that in most real-world settings of binary classification such as criminal risk prediction, it is impossible to satisfy two reasonable measures of fairness (e.g., equal error rates and calibrated predictions) simultaneously.^32^^,^^33^

Even when considering one metric at a time, pursuing metrics can create serious problems. Optimization relies on each metric to be a perfect measure of the unobservable fairness construct being measured.^34^ This metric optimization can then cause problems such as overemphasizing easily measured short-term goals (e.g., clickthrough rate over reported preference)^16^ or leveling-down models (i.e., achieving fairness by making most groups worse off).^35^ Optimization is also likely to push models into edge cases where measurement becomes misdirected (e.g., Goodhart’s law).^36^ For example, a benchmark that productively measures vulgar language, when optimized, may remove innocuous uses of language where individuals reclaim certain slurs.^37^

For these reasons, fairness leaderboards should be abandoned. This, however, does not mean abandoning fairness benchmarks, especially benchmark suites, which can provide a comprehensive and meaningful assessment of AI fairness without reducing findings to a single leaderboard number.

## Benchmark suites for assessing fairness

We argue for the development and use of benchmark suites. Benchmark suites are evolving collections of benchmarks specific to a modality (e.g., computer vision, natural language processing) and particular task that can vary in specificity (e.g., chatbot, algebra tutor chatbot). Application context may change both the composition of the benchmark suite and/or the interpretation of a standardized suite. Suites may be curated or may emerge through common practice and consensus. The validity of benchmark suites should then be assessed not only at the benchmark level but suite level as well. We recommend the use of “suite” instead of “benchmark” to describe these collections, since this conflation in terminology has made it easy to expect one score from a suite and neglect multi-faceted coverage.

Benchmark suites should be accompanied by tools to help visualize and interpret the measurement results. It will likely be important to look into the actual inputs and outputs of a model during this interpretation.^24^ Researchers and practitioners alike can then use these results to assess the prevalence and strength of a set of fairness harms for new models and algorithms. These findings can be used to guide research progress as well as model selection for deployment.

### Making research progress

Leaderboards have been fundamental to making research progress. Without comparing methods based on leaderboard performance, we need a new way to judge progress. We propose progress to take the form of new methods that control trade-offs that we did not have the levers to access before. This would expand the available fairness tools that practitioners can choose from, and in determining which kinds of levers are more valuable, also motivate research progress based on work that grounds fairness in concrete applications.^38^^,^^39^ In our proposal, a fairness researcher fine-tuning an LLM can run a suite of fairness benchmarks to understand what sorts of prompts may lead to discriminatory, stereotyping, or demeaning behavior. Depending on the relevance of the exhibited harms, the researcher can then test a number of methods to understand whether these issues can be mitigated or traded off, or if new techniques should be explored or new training datasets created. A benchmark suite can also help the researcher discover fairness-related harms they may not have thought to look for.

Likewise, a non-fairness AI researcher developing a new algorithm or model may use a fairness suite as a tool for monitoring and reporting unintended fairness consequences. At present, a researcher must individually select which benchmarks to test, and this requires specialized knowledge of the fairness landscape. Moreover, the relevant fairness benchmarks may not be obvious at all. For example, seemingly innocuous changes to model structure can create unexpected social disparities,^40^ as seen with the “truncation trick” for image quality improvement in generative adversarial networks^41^ or even with different computing hardware.^42^ Established benchmark suites would make it easier to discover these impacts at the time of development, without having to anticipate which individual benchmarks should be run for which models. Peer reviewers can then choose to judge the research progress of a particular proposed method with this context about the externality effects of that method.

When using benchmark scores, a popular technique is to make sense of trade-offs using visualizations like Pareto frontier curves and radar plots. Visualizations like these can lead to somewhat arbitrary ways of summarizing results that are just as unfounded as averaging, such as the order of each metric on an axis having a large effect.^43^ Instead, researchers can work to develop visualizations that help identify which benchmarks are correlated or conflicting, and potentially even incorporate friction by design^44^ with respect to quick “top model” selection.

### Selecting a model

When selecting a model for development or deployment, rather than defaulting to the results of a leaderboard, practitioners can use benchmark suites to probe a range of potential fairness impacts of a model and make their own contextualized judgments. Benchmark suites can also help to differentiate in the case of model multiplicity (i.e., when multiple models have equal performance under one metric but differ in other metrics due to differences at the individual prediction level).^45^^,^^46^ Practitioners may often have internal, proprietary benchmarks that can complement public suites. Model cards represent a post-deployment version of this assessment^47^ and provide public disclosure of potential fairness issues.

To actually make concrete choices about models by trading off between benchmarks, we echo others’ recommendations to perform qualitative analyses of quantitative measurements.^16^^,^^48^ These qualitatively determined rankings by experts may even compose a specialized leaderboard for a particular application, when the application context and considerations are well understood. Specialized leaderboards should be explicit about which values were prioritized, and may be helpful for filtering and choosing among many possible models. However, leaderboards should not be used without clear and explicit justification because of human cognitive biases that cause us to favor leaderboard results despite any caveats, thus leading to the problems we discussed.

There are a number of different ways that qualitative analyses of quantitative benchmarks can be performed. Transparent deliberations can make clear whose values are prioritized, which can help make space for deferring to epistemic authorities. The epistemic authority of different stakeholders is relevant in terms of their expertise, relationship to the model (e.g., creator, user, auditor), and also their demographic positionality. Given the history of epistemic injustice,^49^ practitioners should be prepared to elevate the concerns of historically marginalized and ignored groups that may be the most vulnerable.^49^^,^^50^ Overarching incentive structures such as profit may come to bear on these, but we should aim to consider collective ways we can continue to elevate social considerations (e.g., consumer power through market backlash), even when they go against what is profitable.^51^^,^^52^^,^^53^^,^^54^^,^^55^^,^^56^

## Toward more comprehensive benchmark suites

With this vision of AI fairness driven by benchmark suites rather than by leaderboards, a remaining question is whether it is possible to build a sufficiently comprehensive benchmark suite to make this vision a reality. Indeed, developing useful suites is itself an important research problem. A key goal of suite development should be to increase usefulness by expanding (or shrinking) the set of benchmarks included in a suite based on systematic analyses of the coverage, context, and limitations of a suite.^57^

Existing benchmark suites cover multiple facets of what they intend to measure (e.g., trust, capability), but while they aim for comprehensiveness, in practice they are often compiled based on convenience and access. Some, like HELM,^24^ do partially map out the negative space of what is not measured. Other recent work takes an important step by displaying a number of dimensions without foregrounding a leaderboard based on score averages.^9^

Building a good benchmark suite is not easy. We argue that there is more to do toward creating suites that increase both coverage and context. This is true even if a benchmark suite is created without a specific deployment setting in mind, although that certainly changes the importance considerations of the various constituent benchmarks. We therefore identify three major shortcomings in current benchmark suites and propose improvements: adopting modality-specific benchmarks, increasing the coverage of harms, and incorporating diverse perspectives.

### Benchmarks specific to individual modalities

Fairness evaluations originated in binary classification, and comprehensive collections of fairness metrics often focus only on the binary setting.^58^^,^^59^ Given the availability of resources for fairness evaluation in the binary setting, evaluations in other settings often repurpose a benchmark measurement that evaluates one modality (e.g., multiple choice classification) for use in evaluating a different modality (e.g., natural language text, image, video).

Unfortunately, these mismatched evaluations create two problems. First, evaluation findings do not transfer well across modalities.^60^ For example, existing benchmark suites including Claude 3’s model card,^25^ TrustLLM,^23^ and DecodingTrust,^22^ only evaluate fairness in text generation via multiple-choice questions. Claude 3 uses BBQ,^5^ the multiple-choice social bias benchmark explained in the introduction, to measure whether an LLM picks the multiple-choice answer that reinforces a stereotype. However, a model’s behavior on multiple-choice questions is not necessarily predictive of that model’s behavior on natural text output.^60^ Additionally, multiple-choice benchmarks fail to capture text-specific linguistic harms (e.g., person-first versus identity-first language when talking about disability^61^).

This is the second problem: modality-specific harms (e.g., those unique to text data) are missed.^62^ One prominent example is how measuring fairness in image captioning based on the accuracy of a caption’s inferred gender labels^63^ misses potential harms of open-ended text generation and other image-specific concerns.^62^ Another example is evaluation of robotic systems purely in terms of image and textual metrics^64^^,^^65^ (e.g., robots instructed to select among physical blocks with pictures of faces from different racial groups for the “criminal” block^66^), which does not evaluate the unique physical harms possible with real robots.

Hence, improving fairness evaluation requires creating benchmarks specific to individual modalities. These evaluations likely require domain-specific insights from outside of computer science. For example, in image generation the analysis of depicted minority group members being either tokenized or genuinely represented could draw from media studies,^67^^,^^68^ while in text generation, the analysis of stereotypes could leverage theoretical models from social psychology. As an example of operationalizing the latter, the stereotype content model theorizes that all group stereotypes are along the dimensions of warmth and competence (e.g., women are seen to be warm but not competent),^69^ and scholars in social psychology have built dictionaries for measuring these concepts in text.^70^

### Benchmarks for broader ranges of potential harms

AI fairness research has tended to organize around a few notions of harm. Most early work focused on harms resulting from the differential allocation of resources (e.g., jobs, loans). To expand the focus beyond merely allocational harms, researchers introduced representational harms as those that affect the beliefs, and thus standings, of social groups in society^71^ (e.g., search engines overrepresenting White men for “CEO”^72^). Within representational harms, stereotyping has often dominated as the harm of focus.

While this approach has led to valuable research progress, it has also overlooked many other important harms. For example, non-stereotyping representational harms are ignored, like the erasure of social groups (e.g., misrecognizing a hijab as a hoodie in a religious image) or the reification of social groups (e.g., assuming everyone with long hair is a woman).^73^

Additionally, existing benchmarks have often overlooked equity-based harms in favor of equality-based harms.^74^ Popular equality-based benchmarks rely on evaluations grounded in invariance and “color-blindness” (i.e., that each demographic group is treated identically). For example, language benchmarks commonly measure changes in model output when the social group of the input is perturbed.^24^ Strong benchmarks in other domains like computer vision similarly tend to focus on equality-based assessments.^75^ This focus on equal treatment overlooks that certain harms grounded in historical injustice, like misclassifying a person as a gorilla,^76^ are more harmful for individuals of certain racial groups than others. Benchmark suites that ignore equity-based harms fail to account for how social context can cause different groups to experience varying degrees and types of harm.

To develop more comprehensive benchmark suites that better capture the full range of potential harms, we advocate for developing benchmarks based on systematic analyses of coverage, rather than merely ad hoc, and convenient, combinations. Proposed taxonomies of harm can help in this goal by mapping out areas of further benchmark development.^73^^,^^77^^,^^78^ Prior work demonstrates a proof-of-concept for building out a benchmark suite based on a taxonomy of representational harms in image captioning.^62^ In doing so, it is just as important to record the harms not covered by a benchmark suite as it is to record what is covered. Red-teaming, which has its own set of limitations,^80^ can be complementary and help to fill in some holes as well.^79^

In another direction, when thinking about the demographic groups considered, there has been progress in expanding beyond two groups to multiple intersectional ones. However, as a research community, we are only beginning to think about demographic identities that defy traditional categorization like non-binary gender and residual racial categories like “Multiracial” and “Other.”^81^^,^^82^ These groups may experience specific harms (e.g., misgendering, poor reactions to gender disclosure) that are important to include.^83^

### Benchmarks representing different perspectives

Traditional benchmarks usually aggregate ground-truth labels from different human annotators, often by majority vote. However, an annotator’s identity (demographic and otherwise) influences which label they will assign.^84^^,^^85^^,^^86^^,^^87^ For example, stereotypes are culture specific,^88^^,^^89^ natural language text toxicity can vary based on familiarity and comfort with African American Vernacular English,^90^^,^^91^ and experiences of stereotype harm can vary based on gender identity.^92^ Averaging annotations loses these important distinctions.

In fairness, we should explicitly capture the relevant standpoints that individuals come from by virtue of their lived experiences.^93^^,^^94^ In instances where combining annotations results in erasing certain voices, more granular benchmark measurements should be considered that disentangle the tension and make it explicit—in other words, by having separate measurements for each side of the tension. In doing so, we will have to be careful about exploitative practices that overburden members of marginalized groups.^95^ Instead, we can move toward more substantive participatory approaches by bringing in relevant stakeholders far earlier than simply to annotate.^96^^,^^97^ This ranges from determining what should be annotated in the first place, to beyond consultation toward inclusion, collaboration, and ownership as is appropriate.^98^

## Conclusion

Overall, we defend benchmarks but condemn leaderboards in AI fairness. In lieu of leaderboards for making research progress and selecting models, we offer alternative approaches using benchmark suites. This will require improving the coverage and context of benchmark suites by employing modality-specific benchmarks, covering a broader range of harms, and incorporating diverse perspectives. Future work will be needed to assess the missing gaps in benchmark suites, which can motivate the development of even further benchmarks. Benchmark suites are not intended to take the place of ongoing and adaptive system evaluation.^99^ Rather, they are complementary sources of information.

In addition to benchmarks, qualitative assessments will remain critical. This is especially true for groups that are very heterogeneous (e.g., non-binary, Disabled, Indigenous) or may contain too few members to perform robust quantitative analyses (e.g., intersectional identities).^100^^,^^101^

Taking a broader view than just the model level at which benchmarks often measure, developers should remember to still consider system-level interventions.^102^ Systems are broader than models and encompass additional components such as the model interface and how a model is actually used. At the system level, developers can often overcome trade-offs that seemed inevitable at the model level. Let us consider many of the examples we have discussed throughout this paper. In the case of truncation in Generative Adversarial Networks where there is a tension between image quality and diverse racial representation, we could expend resources to collect more images of the underrepresented group and thus improve both image quality and group representation. In the case of image captioning, rather than having a computer vision model either infer gender and risk misgendering someone, or abstain from doing so and thus not provide blind and low-vision users with sufficient context to understand an image fully, the system could prompt self-identification from the depicted individuals and use those identities in any descriptors.^28^ As for criminal risk, instead of predicting who might not appear for their court date and preemptively detaining individuals, text reminders of court dates that serve as behavioral nudges can be effective, eliminating much of the need for AI in this use case.^103^ We can also imagine even broader solutions than these. All interventions should be done in tandem with structural changes that may even obviate the need for the algorithm in the first place, as well as considerations about where abolitionary acts of refusal toward the technology may be more appropriate.^104^

Changing the status quo usage of leaderboards will be difficult, since leaderboards offer an easy-to-understand, collectively established yardstick for model superiority. Nevertheless, embracing a more nuanced approach to benchmarking—and discarding the idea that a single model can be best suited for all tasks—will better equip researchers and practitioners to confront the complexities of the societal implications of AI. In doing so, we can also hope to avoid algorithmic monocultures where the same model being used across domains may arbitrarily exclude the same individuals,^105^^,^^106^ and find ourselves with a far greater diversity of models that better reflect society’s varied needs.

## Acknowledgments

This material is based upon work supported by the 10.13039/100000001National Science Foundation under grant nos. 1763642, 2112562, and 2145198 to O.R. and a Graduate Research Fellowship to A.W., as well as the Microsoft AI & Society Fellowship to A.W. Any opinions, findings, and conclusions or recommendations expressed in this material are those of the author(s) and do not necessarily reflect the views of the 10.13039/100000001National Science Foundation. A.H. is an employee of Adobe Research and is affiliated with the University of Washington. We thank Zoya Bylinskii, Vikram Ramaswamy, and Ye Zhu for helpful feedback and comments.

## Author contributions

All three authors worked together to brainstorm, ideate, and write the paper.

## Declaration of interests

The authors declare no competing interests.
